# Center of mass acceleration during walking: comparison between IMU and camera-based motion capture methodologies

**DOI:** 10.1017/wtc.2024.12

**Published:** 2024-11-22

**Authors:** Jasmine Y. Liang, Li-Shan Chou

**Affiliations:** Department of Kinesiology, Iowa State University, Ames, IA, USA

**Keywords:** gait balance, center of mass, inertia measurement unit, motion capture system

## Abstract

Placing an inertial measurement unit (IMU) at the 5th lumbar vertebra (L5) is a frequently employed method to assess the whole-body center of mass (CoM) motion during walking. However, such a fixed position approach does not account for instantaneous changes in body segment positions that change the CoM. Therefore, this study aimed to assess the congruence between CoM accelerations obtained from these two methods. The CoM positions were calculated based on trajectory data from 49 markers placed on bony landmarks, and its accelerations were computed using the finite-difference algorithm. Concurrently, accelerations were obtained with an IMU placed at L5, a proxy CoM position. Data were collected from 16 participants. Bland–Altman Limits of Agreement and Statistical Parametric Mapping approaches were used to examine the similarity and differences between accelerations directly obtained from the IMU and those derived from position data of the L5 marker (ML5) and whole-body CoM during a gait cycle. The correlation was moderate between IMU and CoM accelerations (*r =* 0.58) and was strong between IMU and ML5 or between CoM and ML5 accelerations (*r =* 0.76). There were significant differences in magnitudes between CoM and ML5 and between CoM and IMU accelerations along the anteroposterior and mediolateral directions during the early loading response, mid-stance, and terminal stance to pre-swing. Such comprehensive understanding of the similarity or discrepancy between CoM accelerations acquired by a single IMU and a camera-based motion capture system could further improve the development of wearable sensor technology for human movement analysis.

## 1. Introduction

The motion of the whole-body center of mass (CoM) reflects the overall mechanical effect on an individual. It has been frequently assessed in the study of human locomotion, especially when examining balance control during standing or walking (Winter 1995; Pai and Patton 1997; Hof 2008). Altered CoM motions have been reported in individuals with gait dysfunction, and many CoM-related kinematic markers have been identified to detect gait imbalance (Kaya et al. 1998; Hahn and Chou 2004) sensitively. Age-related sagittal plane CoM motion reductions were reported during walking and obstacle crossing (Fujimoto and Chou 2014). Increased frontal plane CoM displacement and peak velocity during walking were reported in older fallers (Lee and Chou 2006) and patients with post-concussion syndrome during dual-task walking (Howell et al. 2013). Besides, CoM accelerations were reported to better differentiate between individuals with and without functional limitations (Fujimoto and Chou 2012; Fujimoto and Chou 2014) and suggested to better identify individuals with balance control deficits during daily activities.

The CoM motion is traditionally estimated through the acquisition of whole-body movement with the use of a camera-based motion capture system (Winter 1995), which has been well-developed and regarded as the gold standard. However, practical applications of this method are limited for many real-world activities or clinical settings due to the exorbitant costs associated with equipment and extensive expertise required for data acquisition and analysis. Recent advancements in wearable sensor technology have emerged as a viable solution for effectively bridging this existing gap. Many of these devices incorporate multiple sensors such as tri-axial accelerometers, gyroscopes, and magnetometers and are broadly described as inertial measurement units (IMUs). Such technology provides users substantial freedom to measure body movement and alignment data from various daily activities. Wearable sensors have become a popular alternative in providing a time-efficient and user-friendly measurement of gait and balance performance (O’Sullivan et al. 2009; Pitt and Chou 2019). Recent studies utilizing a single accelerometer placed over the 5th lumbar vertebrae (L5), a proxy for the whole-body CoM, to examine gait balance control concluded that such acceleration data were reliable, clinically practical, and could be sensitive to detect gait imbalance (Howell et al. 2015; Pitt and Chou 2019).

The CoM of a multi-segment system, like the human body, is an estimated point that accounts for each body segment’s mass and instantaneous location. Thus, the CoM location and proximity to selected bony landmarks during movement are posture-dependent. On the other hand, the use of a wearable sensor requires its placement at a fixed body landmark, which does not account for multisegmental motion or any instantaneous changes in body segment alignment that lead to the relocation of the whole-body CoM. Although kinematic parameters derived from the CoM motion and wearable sensor’s measurement have been used to detect gait imbalance, it is still not clear to what extent the acceleration measured by a wearable sensor at a single landmark, i.e., L5, could reflect the feature of the CoM acceleration during walking. Given the growing use of wearable sensors in human movement analysis, such understanding of differences in measurement data from both methods would enhance our ability in IMU data interpretation and the development of deep learning algorithms to facilitate a robust gait imbalance detection.

Therefore, this study aimed to compare the similarity between accelerations directly measured by a single IMU placed at L5 and those derived from the CoM position obtained from a camera-based motion capture approach during walking. This initial study employed healthy adults, a population with a stable gait and balance control, so a fundamental understanding of the agreement between IMU-based measurements and conventionally calculated CoM acceleration could be achieved. It was hypothesized that IMU-based measurements and camera-based CoM acceleration would demonstrate a substantial degree of similarity in the nature of the patterns but with identifiable discrete differences in magnitudes.

## 2. Methods

### 2.1. Participant

A total of 16 healthy adults (8 males/8 females; ages: 19–62 years old; weight: 73.7 ± 18.3 [43.4–106.8] kg, height: 1.71 ± 0.10 [1.56–1.89] m) were recruited for this study from the university community. Individuals with any existing pain or musculoskeletal injuries that could potentially impact walking ability were excluded from the study. Before data collection, participants were provided with a detailed explanation of the study objectives and experimental procedures and signed the informed consent approved by the Institutional Review Board.

### 2.2. Procedure

Forty-nine retroreflective markers were placed on specific bony landmarks (Leardini et al. 2007, 2011) to capture the whole-body motion during walking. Marker trajectory data were collected using a 12-camera motion capture system (Qualisys AB, Sweden). Before data collection, the coordinates of the motion capture system were calibrated and established with one of the axes aligned with the direction of the walkway. Additionally, a single IMU (OPAL, APDM wearable Technologies, Inc., Portland, OR, USA) was placed at the L5 as the proxy location of CoM (Howell et al. 2015). Its axes were aligned carefully with the anatomical direction during a standing position. Moreover, another retroreflective marker was placed on the IMU sensor to acquire movement data at L5 to identify system-wide (IMU versus camera-based) measurement discrepancies.

Participants were instructed to walk at self-selected speeds barefoot along a walkway in the laboratory. To familiarize the laboratory settings and to obtain each participant’s average walking speed, five practice walking trials were first conducted. Marker trajectory and IMU data from each participant were then collected from five subsequent walking trials. The walking speeds for these trials were set within a range of 95% to 105% of the pre-determined average walking speed to ensure the consistency of the gait pattern within each participant.

### 2.3. Data processing

The three-dimensional marker trajectory data were collected with a sampling rate of 240 Hz and processed using a zero-lag low-pass fourth-order Butterworth filter with a cutoff frequency of 12 Hz (Winter 2009; Pitt and Chu 2019). The position of the whole-body CoM was computed as the weighted sum of each body segment’s CoM from a 13-link biomechanical model, including head and neck, trunk, pelvis, two upper arms, two forearms with hands, two thighs, two shanks, and two feet (Hahn and Chou 2004). Anthropometric reference data were adopted from the initial work of Dempster (Dempster 1955). The following equations describe the weighted sum formula for calculating the whole-body CoM position (*x*_0_*, y*_0_*, z*_0_) (Winter 1995).

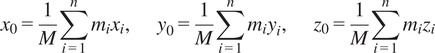

 where *m_i_* is the mass of the *i*th body segment; (*x_i_, y_i_, z_i_*) is the location of the center of mass of the *i*th body segment; and *M* is the total body mass.

Velocities and accelerations for the whole-body CoM and L5 marker, respectively, were calculated from the first and second derivatives of their position data using the finite-difference algorithm built in Visual3D (C-Motion, Inc., MD). IMU acceleration data were collected with a sampling rate of 128 Hz, and raw signals were filtered with a second order, zero-lag, and low-pass Butterworth filter with a 12 Hz cutoff frequency (Hahn and Chou 2004). To mitigate the impact of gravitational acceleration on IMU measurements, the resulting magnitude from the *z*-axis was corrected by subtracting 9.81 m/s^2^.

Data for each gait cycle obtained from both systems were synchronized by aligning the timing markers detected for the gait event of heel-strike (HS). A gait cycle is defined as the time period between two consequtive HSs of the same foot. For the camera-based motion capture system, the HS was determined by the ground reaction force and motion data, utilizing an algorithm integrated in Visual3D. IC events were detected from the IMU acceleration data by detecting the minimum value after applying Gaussian continuous wavelet transforms to the vertical acceleration (McCamley et al. 2012), a process implemented in Matlab (MathWorks, Natick, MA, USA).

### 2.4. Statistical analysis

Correlation between the time-series of acceleration data obtained from the IMU (IMU), a marker placed on the IMU at L5 (ML5), and whole-body CoM over one gait cycle was assessed using the Pearson Correlation Coefficient (*r*). which indicates the degree and direction of the linear relationship The Bland–Altman analysis (Bland and Altman, 1999) was further used to evaluate the level of agreement between measurements. This analytical approach is widely employed in biomedical research to quantify the consistency and repeatability between two measurement techniques (Klein et al. 1998). All data points acquired by the IMU during walking were included in the analysis, as removing any outliers could compromise the true representation of the sensor’s behavior in response to the human walking motion. The analysis created a scatter plot that incorporates the absolute differences between measurements obtained from the IMU and the motion capture system, with the horizontal axis representing the average values of measurements and the vertical axis against corresponding differences. A statistical significance α level was set as 0.05.

To compare the general patterns of IMU, ML5, and CoM accelerations over one gait cycle, a one-way repeated analysis of variance (ANOVA) was conducted with the normalized acceleration data using the Statistical Parametric Mapping (SPM) approach. The data normalization process involved scaling the maximum or minimum value to fit within the range of −1 to 1. A statistical significance α level was set as 0.05. Post-hoc pairwise comparisons were performed with a significant α level of 0.017 with Bonferroni correction. Data analyses were conducted using MATLAB, and the statistical analyses were implemented in spm1d (Pataky 2012; Pataky et al. 2016).

## 3. Results

**Figure 1.**
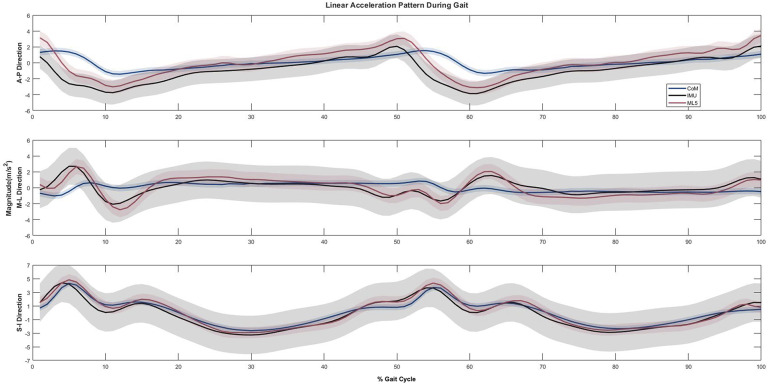
Average accelerations from all participants were obtained by different approaches (CoM, IMU, and ML5) in three anatomical directions during one gait cycle. The grey shade in the plot, represented by the ±1 standard deviation (SD), indicates the dispersion or variability of the data points around the mean. A gait cycle is defined as ranging from an initial contact (0%) to the subsequent initial contact (100%) of the same foot. Anteroposterior (A-P), mediolateral (M-L), and superior–inferior (S-I).

Accelerations in three respective anatomical directions obtained from all three approaches demonstrated similar patterns (Figure 1). Acceleration data directly measured by the IMU closely overlapped with that of ML5 in all three directions. However, CoM accelerations had relatively lower magnitudes. They were smoother when compared to those of IMU and ML5, especially at the heel-strike and mid-stance, and such a phenomenon was more noticeable in the mediolateral direction.

**Figure 2.**
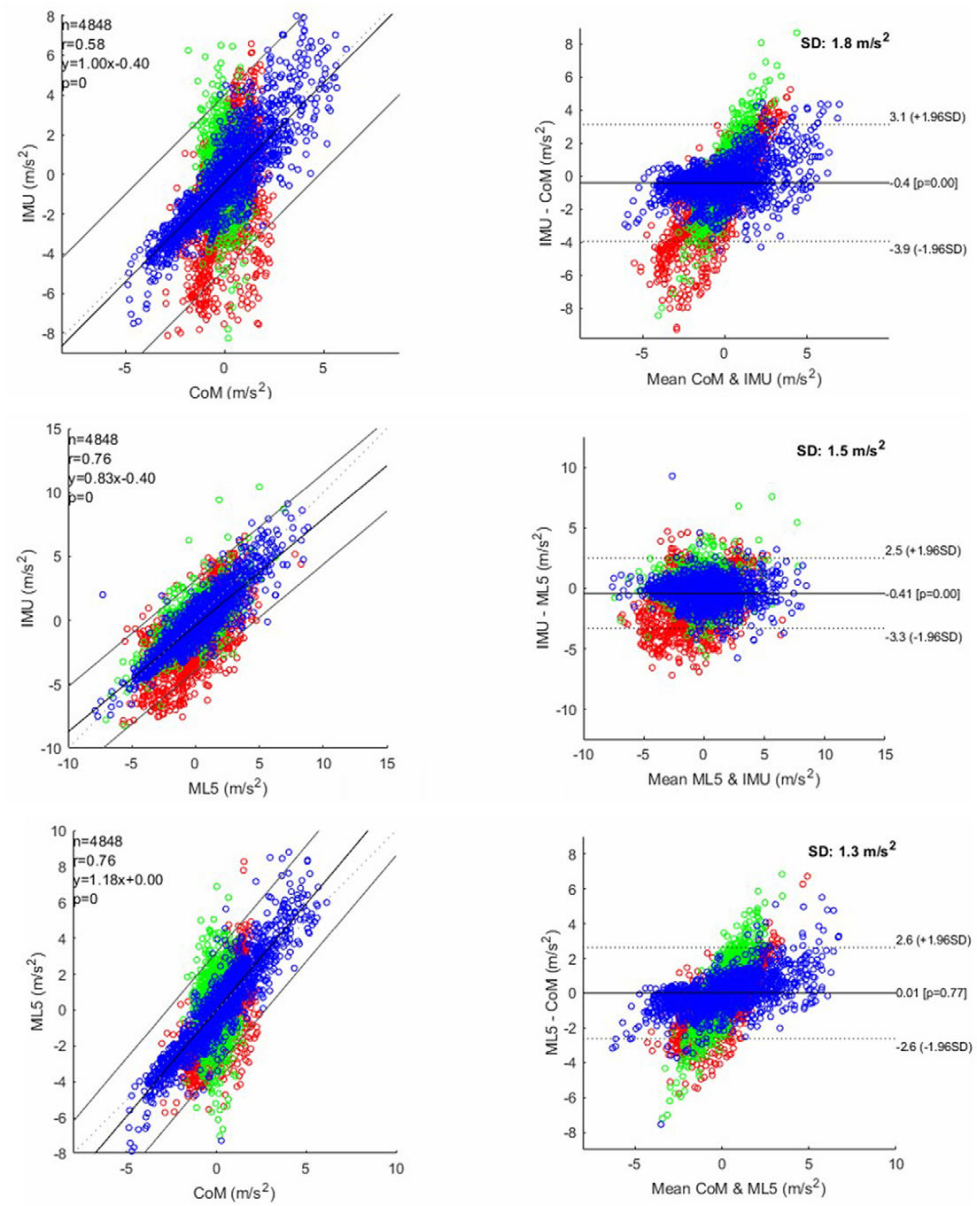
Linear correlation analysis (left-column) and Bland–Altman plot (right-column) between each pair of measurements: IMU versus CoM; IMU versus ML5; ML5 versus CoM. (red: anteroposterior; green: mediolateral; blue: superior–inferior).

A total of 4848 points were included in both correlation and Bland–Altman analysis (16 participants × 101 timeframes × 3 directions). The correlation was moderate between IMU and CoM (r = 0.58; p < .05) and was strong between IMU and ML5 or between CoM and ML5 (r = 0.76; p < .05) (Figure 2). The Bland–Altman plot quantified the biases and variabilities for three pairs: CoM versus IMU, ML5 versus IMU, and CoM versus ML5. The upper and lower limits were calculated using mean ± 1.96 × standard deviation (𝑆𝐷). For the comparison between CoM and IMU, the absolute mean bias ± 𝑆𝐷 was −0.4 ± 1.8 m/s^2^, with the limits of agreements being −3.9 and 3.1. For the comparison between ML5 and IMU, the absolute mean bias ± 𝑆𝐷 was −0.41 ± 1.5 m/s^2^, with the limits of agreements being −3.3 and 2.5. For the comparison between CoM and ML5, the absolute mean bias ± 𝑆𝐷 was 0.01 ± 1.3 m/s^2^, with the limits of agreements being −2.6 and 2.6. (Figure 2).

**Figure 3.**
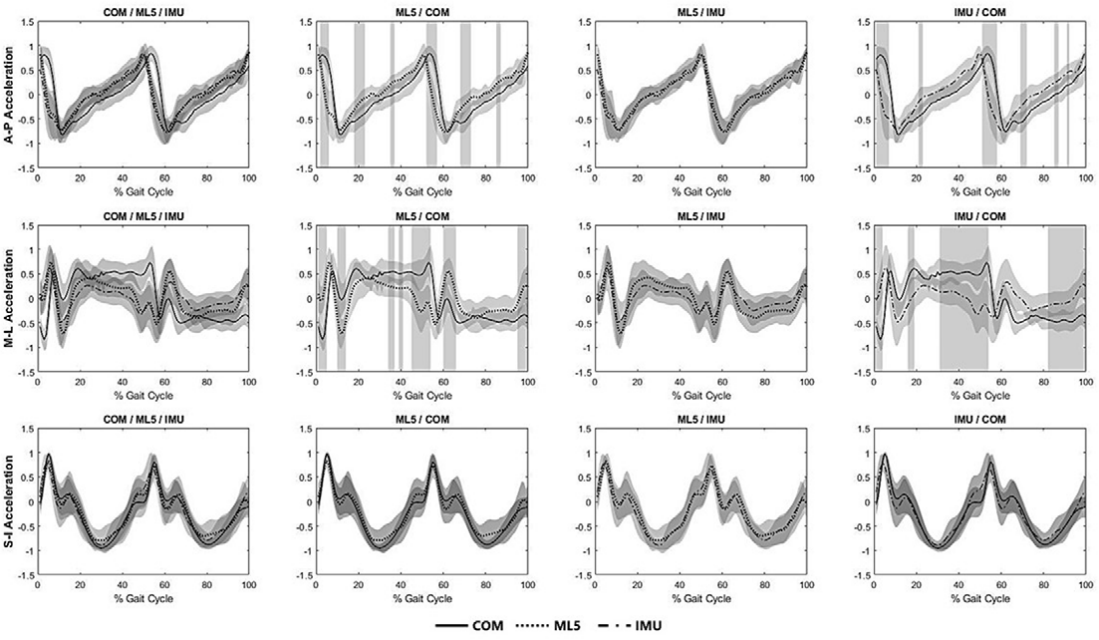
Normalized accelerations (with ±1 SD in shaded grey) from IMU, CoM, and ML5 in each of three anatomical directions during a gait cycle. Regions with statistical significances from the SPM one-way repeated ANOVA were indicated in grey color zones.

When comparing the normalized magnitudes, significant differences were detected between IMU versus CoM and ML5 versus CoM in both anteroposterior and mediolateral directions (Figure 3). In the anteroposterior direction, significant differences were detected between CoM and IMU and between CoM and ML5 during the loading response, mid-stance, and late terminal stance (*p <* .001). In the mediolateral direction, there were significant differences between CoM and ML5 at the heel-strike, early mid-stance, and late terminal stance phase (*p <* .001) and between CoM and IMU at the early loading response, mid-stance, and terminal stance to pre-swing (*p <* .001).

## 4. Discussion

We examined to what extent the accelerations measured by an IMU placed at the L5 could reflect the feature of the whole-body CoM accelerations during walking. Our findings indicated that the accelerations measured at the L5 with an IMU or with a single marker demonstrated similar patterns in all three anatomical directions to the CoM accelerations derived from multi-segment kinematic data obtained with a camera-based motion capture system. However, our results also indicated the presence of a systematic and proportional bias between accelerations measured by IMU and derived from CoM position data. Such systematic bias is more pronounced in the mediolateral and anteroposterior directions, especially during the loading response and terminal stance.

Accelerations obtained from a single marker placed at L5 with IMU were also included for comparison in this study. As accelerations of the L5 marker were calculated using the same method as for the whole-body CoM, its comparison to the IMU-measured acceleration would reveal possible differences resulting from the numerical method applied to calculate the second derivatives from position data. The close agreement between the accelerations obtained from the marker and IMU placed at L5 indicates that the acceleration computation from position data would not be a contributing factor to the magnitude difference between IMU-measured and motion capture system-derived CoM accelerations.

Analysis from the Bland–Altman plot revealed essential insights from the IMU and CoM comparison and indicated the presence of a systematic and proportional bias between accelerations measured by IMU and derived from CoM position data. The whole-body CoM of a multi-segment system is estimated using the weighted-sum method that accounts for masses and instantaneous locations of all body segments. Its kinematics reflects the overall mechanical effect of the multi-segment system. During walking, the whole-body CoM trajectory is smoothly and tightly regulated to pass between the alternating supporting feet (Winter 1995; Chou et al. 2001). It is, therefore, reasonable to expect a smoother CoM acceleration profile compared to those obtained from the marker or IMU placed at L5, which only measures the kinematics of the pelvis segment. Such systematic bias is more pronounced in the mediolateral and anteroposterior directions, in which the CoM motion is tightly regulated in response to changes in the base of support, especially during the loading response and terminal stance. Despite differences in the acceleration magnitude, both the CoM acceleration obtained with the camera-based system and acceleration measured at the L5 by an IMU were reported to detect gait imbalance (Pitt and Chou 2019). However, such a comprehensive understanding of differences in measurement data from both methods would enhance our ability in IMU data interpretation and the development of deep learning algorithms to facilitate robust gait imbalance detection.

### 4.1 Limitation

There were a few potential limitations in this study. First, during data collection, the local IMU coordinate system was not explicitly aligned with the global coordinate system. Although the participant’s walking direction was guided by the walkway that aligned with one of the axes established for the camera-based motion capture system and the IMU at L5 was also oriented carefully with the anatomical direction during a standing position, this setup could still introduce directional discrepancies in respective data measurement. Such limitation may impose a greater difference in activities involving a significant trunk movement. Secondly, this study is our initial investigation to examine the data agreement between the uses of IMU and camera-based motion capture systems for estimating CoM acceleration. As a result, only healthy adults were recruited as participants. Future studies should consider examining whether the reported data differences vary with different locomotive tasks or populations, including older adults or individuals with impaired balance, to enhance the generalizability of findings.

In addition, the body sizes of study participants may impact the CoM calculation and IMU placement, which could lead to a potential variation in the acceleration measurement. This current study did not explicitly address how these variations may influence the results. Future efforts should be made to investigate these relationships more thoroughly, providing a more comprehensive understanding of the implications of varying body sizes on interpreting CoM acceleration data obtained through IMUs.

## 5. Conclusion

This study aimed to assess the agreement between accelerations measured by an IMU placed at L5 and whole-body CoM accelerations calculated by a camera-based motion capture system during walking. The results revealed congruent patterns in both systems across all three anatomical directions but noticeable differences in magnitudes during specific gait phases. Bland–Altman analysis indicated a systematic agreement bias between the two systems, and the disparity was unrelated to the employed acceleration computation algorithm. A smoother CoM acceleration profile reflects its unique kinematic characteristics as an equilibrium point of the multi-segment human body.

Such comprehensive understanding of the similarity or discrepancy between CoM accelerations acquired by a single IMU and a camera-based motion capture system could further improve the development of wearable sensor technology for human movement analysis. Future investigations should focus on refining data acquisition methodologies to mitigate these identified biases or exploring advanced machine learning algorithms or sensor fusion techniques to ensure the capture of relevant kinematics features. These efforts will enhance the accuracy and applicability of IMU-based assessments in the context of whole-body CoM dynamics of human movement.

## Data Availability

All data reported in this study were collected from human subjects tested in the Biomechanics Laboratory in the Department of Kinesiology at the Iowa State University.
